# Supplementary material for: Local and systemic responses to SARS-CoV-2 infection in children and adults
Source: Nature. 2021 Dec 22;602(7896):321–7. doi: 10.1038/s41586-021-04345-x (PMC8828466; doi:10.1038/s41586-021-04345-x)
Supplement: Supplementary file 1 — Detailed cell type annotation, cell type abbreviations for airway and blood immune cells, IFN production in dendritic cells and additional references. [file 41586_2021_4345_MOESM1_ESM.pdf]

nature portfolio

https://doi.org/10.1038/s41586-021-04345-x

Supplementary information

Local and systemic responses to SARS-CoV-2 infection in children and adults

In the format provided by the authors and unedited

Supplementary information

Local and systemic responses to SARS-CoV-2 infection in children and adults

Masahiro Yoshida, Kaylee B. Worlock, Ni Huang, Rik G.H. Lindeboom et al.

Supplementary Information

Detailed Cell Type Annotation

The epithelial cell populations fall into two broad domains, one comprising ciliated cells and a second covering the basal to secretory cell differentiation pathway, as visualised using Velocyto (**Fig. 1d**). These include basal, cycling basal, secretory, goblet and squamous cells (markers in **Extended Data Fig. 2c**). In addition, *KHDRBS2* marked a distinct basal 1 population, whereas basal 2 cells are high in *DAPL1* and *NOTCH1*. Secretory cells express secretory proteins such as mucins and antimicrobial peptides and may be differentiation intermediates giving rise to more differentiated club cells (*SCGB1A1, SCGB3A1). GALNT4* marks a distinct secretory subtype. Goblet cells have high *MUC5AC* expression and were subdivided into *TFF1*-hi goblet 1 and three distinct goblet 2 populations (*BPIFA*2, *PLAU*, and goblet 2 inflammatory cells). Furthermore, we detect squamous cells (*SPRR3*, *KRT78)* and identify three Hillock-like populations[14,26,27], all marked by *KRT14*, *KRT6A* and *KRT13* expression and referred to as Hillock, cycling Hillock and Hillock precursor cells, which form a distinct differentiation trajectory (**Fig. 1d**) similar to the one reported in mouse[27]. Within the ciliated cell domain, the differentiation trajectory points from ciliated 1 (*PIFO*, *OMG*) to ciliated 2 (*CFAP54*, *DZIP1L*) cells. Between secretory and ciliated clusters, we observe deuterosomal cells as intermediates marked by *CDC20* and *FOXN4*[26,33] (**Fig. 1b, d**). Additionally, we detect two novel cell populations that form a second bridge between the secretory and ciliated clusters, which we named Transit epi 1 and 2. They co-express ciliated cell markers (*PIFO*) and secretory genes (*MUC2*), but are *FOXJ1* low. Distinguishing markers for these two clusters are two long non-coding RNAs, *FP671120.*4 and *FP236383.2* for both and *HIST1H1E* for Transit epi 2. Compared to previously described differentiation intermediates from the nasopharynx[14,28], transit epithelial cells express described marker genes at relatively low levels, but do show similarities to IRC[28], secretory-diff cells[28] and SERPINB11-hi secretory cell types[14] (**Extended Data Fig. 3b-d**), although exact gene expression signatures will depend on sampling method, location and clinical covariates. We detect Transit epi 1 mostly in COVID-19 patients, but also in healthy children (**Extended Data Fig. 2a**), suggesting a function in development and tissue regeneration (see below). We also detect rare cell types such as ionocytes, brush cells, neuroendocrine cells, and melanocytes, each expressing their canonical markers[26,33,82,83].

Cell type abbreviations for airway and blood immune cells

Airway immune: Mac: macrophages, LC: Langerhans cells, Mono: monocytes, Neut: neutrophils, DC: dendritic cells, cDC: conventional DC, pDC plasmacytoid DC, fDC: follicular DC, T reg: T regulatory, T fh: follicular helper T cells, mem: memory, MAIT: mucosal-associated invariant T cells, T gd: gamma-delta T cells, NK: natural killer cells, ILC: innate lymphoid cells. B mem: memory B cells, IgK: immunoglobulin kappa, IgL: immunoglobulin lambda.

Blood Immune: CTL: cytotoxic T lymphocyte, CM: central memory, EM: effector memory, EMRA: effector memory re-expressing CD45RA, g/d: gamma-delta, reg: regulatory, MAIT: mucosal-associated invariant T cells, NK: natural killer, NKT: natural killer T, ILC: innate lymphoid cells, Mono: monocyte, pDC: plasmacytoid dendritic cells, cDC: conventional

dendritic cells, AS-DC: AXL+ SIGLEC6+ dendritic cells, n-sw mem: non-switched memory, sw mem: switched memory, invar: invariant, HPC: haematopoietic progenitor cell, Baso/Eos: basophil / eosinophil, RBC: red blood cell, IFN stim: interferon stimulated, Tem; T effector memory.

IFN production in dendritic cells

To identify IFN producing cells that initiate the local and systemic immune response against COVID-19, we ranked all donors by the percentage of IFN-stimulated cells in blood and visualised their global IFN activation and IFN production signatures in nasal resident DCs (**Fig. 3h**, expression of individual genes in all cell types in **Extended Data Fig. 8a**). We observed that individuals with high numbers of IFN-stimulated cells in blood also had high expression of IFN responsive genes in the nose, suggesting that this is where IFN production first occurs. Strikingly, in nasal resident DCs of the highest ranked patient, we observed strong IFN type I and type III production. Examining all cell types of this individual revealed that both pDCs and cDCs, but not any of the other immune or epithelial cells, are producing high amounts of multiple type I and type III molecules (**Extended Data Fig. 8b**). Notably, this asymptomatic individual initially tested negative for SARS-CoV-2 by PCR, followed by a positive test and subsequent sampling within four days, which was validated by the high amount of SARS-CoV-2 reads (**Fig. 1e**). Therefore, sampling occurred at a very early stage of infection, several days before most COVID-19 patients develop symptoms[84]. It is thus likely that we captured the initiation of the immune response via IFN type I and type III signaling at the site of infection. Together, this suggests a key role for nasal DCs as initiators of the immune response against SARS-CoV-2 infection via IFN signaling, and underscores the importance of temporal resolution when studying COVID-19.

Supplemental Note References:

82. Zak, F. G. & Lawson, W. The presence of melanocytes in the nasal cavity. *Ann. Otol. Rhinol. Laryngol.* **83**, 515–519 (1974).

83. Ewing, E. Malignant Melanoma Arising in Association with Sinonasal Melanosis: A Case Report and Review of the Literature. *Int J Pathol Clin Res* **3**, 058 (2017).

84. Tindale, L. C. *et al.* Evidence for transmission of COVID-19 prior to symptom onset. *Elife* **9**, (2020).
