# Supplementary material for: Local and systemic responses to SARS-CoV-2 infection in children and adults
Source: Nature. 2021 Dec 22;602(7896):321–7. doi: 10.1038/s41586-021-04345-x (PMC8828466; doi:10.1038/s41586-021-04345-x)
Supplement: Supplementary file 2 — Reporting Summary [file 41586_2021_4345_MOESM2_ESM.pdf]

nature portfolio

Corresponding author(s): Kerstin B Meyer, Marko Z Nikolic

Last updated by author(s): Nov 9, 2021

Reporting Summary

Nature Portfolio wishes to improve the reproducibility of the work that we publish. This form provides structure for consistency and transparency in reporting. For further information on Nature Portfolio policies, see our Editorial Policies and the Editorial Policy Checklist.

Statistics

For all statistical analyses, confirm that the following items are present in the figure legend, table legend, main text, or Methods section.

n/a	Confirmed	
☐	☒	The exact sample size (n) for each experimental group/condition, given as a discrete number and unit of measurement
☐	☒	A statement on whether measurements were taken from distinct samples or whether the same sample was measured repeatedly
☐	☒	The statistical test(s) used AND whether they are one- or two-sided<br>*Only common tests should be described solely by name; describe more complex techniques in the Methods section.*
☐	☒	A description of all covariates tested
☐	☒	A description of any assumptions or corrections, such as tests of normality and adjustment for multiple comparisons
☐	☒	A full description of the statistical parameters including central tendency (e.g. means) or other basic estimates (e.g. regression coefficient) AND variation (e.g. standard deviation) or associated estimates of uncertainty (e.g. confidence intervals)
☐	☒	For null hypothesis testing, the test statistic (e.g. F, t, r) with confidence intervals, effect sizes, degrees of freedom and P value noted<br>*Give P values as exact values whenever suitable.*
☐	☒	For Bayesian analysis, information on the choice of priors and Markov chain Monte Carlo settings
☐	☒	For hierarchical and complex designs, identification of the appropriate level for tests and full reporting of outcomes
☐	☒	Estimates of effect sizes (e.g. Cohen's d, Pearson's r), indicating how they were calculated

Our web collection on statistics for biologists contains articles on many of the points above.

Software and code

Policy information about availability of computer code

Data collection

No specific code was used in the data collection

Data analysis

The following open access algorithms were used in the data analysis.
Azimuth
bbknn 1.3.12
bedtools v.2.30
Cell Ranger 3.0.2
EmptyDrops
g:profiler toolkit
Harmony
Kraken 2
Scanpy 1.6.0
Scirpyy
Scrublet 0.2.1
Scanpy 1.6.0
scvelo 0.2.2
Seuratt
SoupX 1.5.0 and 1.4.8 as specified in methods
SouporCell
STARsolo functionality of STAR 2.7.3

All data analysis scripts are available on https://github.com/Teichlab/COVID-19paed.

For manuscripts utilizing custom algorithms or software that are central to the research but not yet described in published literature, software must be made available to editors and reviewers. We strongly encourage code deposition in a community repository (e.g. GitHub). See the Nature Portfolio guidelines for submitting code & software for further information.

Data

Policy information about availability of data

All manuscripts must include a data availability statement. This statement should provide the following information, where applicable:

- Accession codes, unique identifiers, or web links for publicly available datasets
- A description of any restrictions on data availability
- For clinical datasets or third party data, please ensure that the statement adheres to our policy

Data availability:
The data set from our study can be explored interactively through a web portal: https://covid19cellatlas.org. Quality control metrics for our single cell data can be found at the web portal page. The data object, as a h5ad file, can also be downloaded from the portal page. The UK data set is available under accession number EGAD00001007718. Counts matrices from bronchial brushings obtained from patients at Northwestern Memorial Hospital, Chicago, are available at GEO, accession number GSE168215. As data is from living patients, these data will be available under managed data access.

The EGA link is:
https://urldefense.proofpoint.com/v2/url?u=https-3A__ega-2Darchive.org_datasets_EGAD00001007718&d=DwIDaQ&c=D7ByGjS34AllFgecYw0iC6Zq7qlm8uclZFI0SqQnqBo&r=UkvGllMAxxOrRLImmtb8_8aL9f8dRmw6ZZOconDDoI&m=ms4g_hTiCC1177yddG023CrSIQfvZR3LHJ-3aHcbNfLqrMJ30dvc2iSSkzVsMJH2&s=yCvFfXAlnXSAk41YM7Fn2afxwbaPZxTYYJDExRQGVLA&e=

The applicant requests specific dataset access via : https://www.sanger.ac.uk/legal/DAA/MasterController

Field-specific reporting

Please select the one below that is the best fit for your research. If you are not sure, read the appropriate sections before making your selection.

☒ Life sciences ☐ Behavioural & social sciences ☐ Ecological, evolutionary & environmental sciences

For a reference copy of the document with all sections, see nature.com/documents/nr-reporting-summary-flat.pdf

Life sciences study design

All studies must disclose on these points even when the disclosure is negative.

Sample size	No sample size calculations were carried out. The following statement was sent to the reviewers:<br>Due to the complexity of single-cell datasets, there are not yet any widely accepted methods available to perform power calculations for studies such as ours. However, the statistical framework that is employed to perform cell type composition analyses in this study specifically fits random effects to model any unexplained variance in a rigorous manner.<br>Single cell sequencing is a technique that gives great in depth insight, but at high financial cost. The total number of patients enrolled in this study was 93, which is in line with or larger than comparable recent studies (see references 10, 11, 12 and 14 in the manuscript).
Data exclusions	All samples for which sequencing data was generated have been submitted to EGA. For the airway data set, 7 samples were excluded from analysis, out of which 1 (AP13-NB) had almost no reads at all, 4 (AN2-NB, AN3-NB, AN7-NB, PP14-NB) had too few reads, 1 (PP7-NB_v2.0) had low mapping rate, and 1 (PC21-NB) failed cell calling. For the PBMC data set, PC7 was of insufficient quality and therefore not included in the analysis.
Replication	All findings were based on statistical analysis of a large patient cohort. There was no replication cohort.
Randomization	As this was not a clinical trial, randomisation was not relevant for our study.
Blinding	As this was not a clinical trial, blinding was not relevant as the statistical tests were performed in a single analysis with all relevant samples included.

Reporting for specific materials, systems and methods

We require information from authors about some types of materials, experimental systems and methods used in many studies. Here, indicate whether each material, system or method listed is relevant to your study. If you are not sure if a list item applies to your research, read the appropriate section before selecting a response.

Materials & experimental systems

n/a	Involved in the study	
☐	☒	Antibodies
☒	☐	Eukaryotic cell lines
☒	☐	Palaeontology and archaeology
☒	☐	Animals and other organisms
☐	☒	Human research participants
☐	☒	Clinical data
☒	☐	Dual use research of concern

Methods

n/a	Involved in the study	
☒	☐	ChIP-seq
☒	☐	Flow cytometry
☒	☐	MRI-based neuroimaging

Antibodies

Antibodies used

anti-human S100A9 conjugated to FITC (clone: MRP14, Biolegend cat. # 350703); anti-human EpCam conjugated to APC (clone: 9C4, Biolegend cat # 324207); 192 TotalSeq-C antibodies (Biolegend, cat. # 99814). The latter was a pre-diluted commercial panel.
S100A9 validation:
Recombinant human S100A8 protein (Cat. No. 719902, lane 1), S100A9 protein(lane 2) and total lysates (15 µg protein) from HeLa (negative control, lane 3) , PBMC (lane 4) were resolved by 4-20% Tris-Glycine electrophoresis, transferred to nitrocellulose, and probed with 1:5000 (0.1 µg/mL) diluted purified anti-MRP-14 (S100A9) (clone A10105J). Proteins were visualized by chemiluminescence detection using 1:3000 diluted HRP anti-mouse-IgG secondary antibody (Cat. No. 405306). 1:2000 dilution of Direct-Blot™ HRP anti-β-actin antibody (clone 2F1-1, Cat. No. 643807) was used as a loading control (lower). Lane M: Molecular weight ladder. The electrophoresis gel shows clear staining in lane2 and 4, but not lane 1 and 3.
validation of EpCam:
Each lot of this antibody is quality control tested by immunofluorescent staining with flow cytometric analysis. For flow cytometric staining, the suggested use of this reagent is ≤ 0.5 µg per 106 cells in 100 µL volume or 100 µL of whole blood. It is recommended that the reagent be titrated for optimal performance for each application.

Validation

All antibodies employed were commercial antibodies.

Human research participants

Policy information about studies involving human research participants

Population characteristics

Population characteristics are listed in Extended Data Table 1.

Recruitment

Recruitment of patients was in line with research ethics permissions listed below. Experienced clinicians assessed each patient and exclusion criteria noted in the methods were applied.

Ethics oversight

Ethical approval was given through the Living Airway Biobank, administered through UCL Great Ormond Street Institute of Child Health (REC reference: 19/NW/0171, IRAS project ID 261511, North West - Liverpool East Research Ethics Committee), REC reference 18/SC/0514 (IRAS project 245471, South Central - Hampshire B Research Ethics Committee) administered through University College London Hospitals NHS Foundation Trust and REC reference 18/EE/0150 (IRAS project ID 236570, East of England - Cambridge Central Research Ethics Committee) administered through Great Ormond Street Hospital NHS Foundation Trust, REC reference 08/H0308/267 administered through Cambridge University Hospitals NHS Foundation Trust, as well as by the local R&D departments at all hospitals. All study participants or their surrogates provided informed consent.

Ethical approval for sample collection from patients with severe pneumonia was given by Northwestern Institutional Review Board, study STU00204868 (PI Richard Wunderink). Samples from patients with COVID-19, viral pneumonia and other pneumonia, and non-pneumonia controls were collected from participants enrolled in the Successful Clinical Response in Pneumonia Therapy (SCRIPT) study STU00204868 and admitted to the ICU at Northwestern Memorial Hospital, Chicago.

Note that full information on the approval of the study protocol must also be provided in the manuscript.

Clinical data

Policy information about clinical studies
All manuscripts should comply with the ICMJE guidelines for publication of clinical research and a completed CONSORT checklist must be included with all submissions.

Clinical trial registration

na

Study protocol

Note where the full trial protocol can be accessed OR if not available, explain why.

Data collection

Describe the settings and locales of data collection, noting the time periods of recruitment and data collection.

Outcomes

Describe how you pre-defined primary and secondary outcome measures and how you assessed these measures.
